# Metformin Promotes Neuronal Differentiation via Crosstalk between Cdk5 and Sox6 in Neuroblastoma Cells

**DOI:** 10.1155/2019/1765182

**Published:** 2019-02-19

**Authors:** Thunwa Binlateh, Supita Tanasawet, Onnicha Rattanaporn, Wanida Sukketsiri, Pilaiwanwadee Hutamekalin

**Affiliations:** ^1^Department of Physiology, Faculty of Science, Prince of Songkla University, Hat-Yai, Songkhla 90112, Thailand; ^2^Department of Anatomy, Faculty of Science, Prince of Songkla University, Hat-Yai, Songkhla 90112, Thailand; ^3^Department of Biochemistry, Faculty of Science, Prince of Songkla University, Hat-Yai, Songkhla 90112, Thailand; ^4^Department of Pharmacology, Faculty of Science, Prince of Songkla University, Hat-Yai, Songkhla 90112, Thailand

## Abstract

Metformin has recently emerged as a key player in promotion of neuroblastoma differentiation and neurite outgrowth. However, molecular mechanisms of how metformin promotes cellular differentiation have not yet been fully elucidated. In this study, we investigated how metformin promotes cell differentiation, via an inhibition of cell proliferation, by culturing SH-SY5Y neuroblastoma cells with or without metformin. Pretreatment with reactive oxygen species (ROS) scavenger, NAC, revealed that ROS plays a crucial role in induction of cell differentiation. Cell differentiation was observed under various morphological criteria: extension of neuritic processes and neuronal differentiation markers. Treatment with metformin significantly increased neurite length, number of cells with neurite, and expression of neuronal differentiation markers, *β*-tubulin III and tyrosine hydroxylase (TH) compared with untreated control. Further investigation found that metformin significantly decreased Cdk5 but increased Sox6 during cell differentiation. Analysis of the mechanism underlying these changes using Cdk5 inhibitor, roscovitine, indicated that expressions of Cdk5 and Sox6 corresponded to metformin treatment. These results suggested that metformin produces neuronal differentiation via Cdk5 and Sox6. In addition, phosphorylated Erk1/2 was decreased while phosphorylated Akt was increased in metformin treatment. Taken together, these findings suggest that metformin promotes neuronal differentiation via ROS activation through Cdk5/Sox6 crosstalk, relating to Erk1/2 and Akt signaling.

## 1. Introduction

Cell differentiation is a process by which nonspecialized cells have become specialized mature cells. Cell differentiation has been widely studied because it can be used as an alternative therapeutic treatment such as degeneration of neuronal cells [1]. SH-SY5Y neuroblastoma cell is considered as* in vitro* models of neuronal function and differentiation because SH-SY5Y can differentiate into mature neuron-like phenotype characterized by neuronal markers [2, 3]. Normally, cell differentiation plays a remarkable inverse association with cell proliferation [4]. A relation between cell proliferation and cell differentiation is observed in G1 phase, regulated by Cdk-cyclin activity and the differentiation induced by transcription factors [5]. Several studies have reported that Akt and Erk signaling pathways mediate regulation of cell differentiation and cell proliferation [6, 7]. However, the mechanism which controls cell differentiation is still not well understood. Several lines of evidence indicate that ROS influences cell differentiation [8, 9]. Differentiation of embryonic stem cell is increased by the induction of ROS via upregulation of gene expression related to mitochondrial metabolic pathway [10]. ROS mediated neurogenesis via different pathway such as the activation of JNK signaling [11] and Wnt/*β*-catenin pathway [12].

Sox6 is a transcription factor in Sox family expressed in neurons [13]. Sox6 is essential for positioning and maturating of cortical interneurons. Mutation of Sox6 in cortical neurons displays immature physiological feature [14]. Sox6 is regulated by Cdk5, a proline-directed serine/threonine protein kinase which functioned in normal and pathological condition of brain cells [15], during neuronal development [16]. Primary cortical neuron transfected with wild-type Cdk5 showed a reduction of Sox6 expression levels while the levels of Sox6 were increased in dominant negative Cdk5.

Metformin is a widely used drug for treatment of diabetic type II patient. Recently, it has been reported that metformin has a potential role in an induction of neural differentiation [17, 18] and neural repair [19]. Antiproliferation effect of metformin induces cell cycle arrest [20, 21], which is mediated by activation of Erk1/2 and Akt [22]. However, the role of metformin in neuronal differentiation has not yet been thoroughly demonstrated. Therefore, the aim of this study is to investigate the mechanism by which metformin promotes cell differentiation in SH-SY5Y neuroblastoma cells.

## 2. Materials and Methods

### 2.1. Reagents

Metformin was purchased from Apex Biotech Company (Taiwan). The Cdk5 inhibitor (roscovitine; Rosc) and ROS scavenger (N-acetyl cysteine; NAC) were obtained from Sigma-Aldrich (St Louis, MO, USA).

### 2.2. Cell Culture

SH-SY5Y neuroblastoma cell line was purchased from ATCC (Bethesda, MD, USA). SH-SY5Y cells were cultured in Dulbecco's modified Eagle's media (DMEM) supplemented with 10% heat-inactivated fetal bovine serum (FBS), 1% L-glutamine and 100 U/ml penicillin/streptomycin (all from Gibco, Grand Island, NY, USA) and incubated in a humidified atmosphere of 95% under 5% of CO_2_ at 37°C. In differentiation studies, the medium was changed to DMEM containing 1% FBS, 1% L-glutamine and 100 U/ml penicillin/streptomycin (starvation culture medium) with or without metformin.

### 2.3. Cell Viability

To examine an effective dose of metformin on SH-SY5Y cells, we performed MTT assay to measure cell viability SH-SY5Y cells were plated on 96-well culture plate (1.5 × 10^4^ cells per well) and incubated for 24 h. Cells were then treated with various doses of metformin (0.5, 1, 5, 10, and 20 mM) for 24 h and with different times (3, 6, 12, and 24 h) at 5 mM in starvation culture medium. At the end of treatment, cells were incubated with 5 mg/ml of MTT (Sigma Chemical CO, St Louis, MO, USA) for 3 h. DMSO was added and the optical densities were measured at 540 nm. The comparative results of cell viability were shown as a percentage ratio with the control untreated group.

### 2.4. Neuronal Morphology Observation and Measurement

To determine the effect of metformin on neuronal morphological change of SH-SY5Y cells, cells were incubated in starvation culture medium containing 1, 5, and 10 mM metformin. Cell morphological changes were captured using Bright-field Olympus Inverted Microscope IX73 (model U-LH100L-3) in comparison between before and after exposure to metformin. The morphological criteria, including the neurite length and numbers of cell with neurite per total cells, were analyzed using Image J software. Measurement of neurite outgrowth was modified from Fado et al. and Chen et al. [23, 24]. Briefly, the neurite length was determined by measuring the total length of the outgrowing neurites per soma. The numbers of cell with neurites which generated longer than twofold of soma's diameter were counted and compared to number of total cells. In all experiments, the data were analyzed from 5 independent random areas. Quantitative data of all morphologies were reported as a comparative percentage ratio with the control untreated cells.

### 2.5. Intracellular ROS Measurement

To investigate the effect of metformin on intracellular ROS production, DCFH-DA fluorimetric assay was performed in order to detect intracellular ROS levels. SH-SY5Y cells were plated on 96-well culture plate (1.5 × 10^4^ cells per well) and incubated for 24 h. Cells were then treated with or without 5 mM metformin for 1, 3, 6, 12, and 24 h. After treatment, media was removed and PBS containing 50 *μ*M DCFH-DA was added for 1 h. The fluorescence densities were detected using an excitation at 485 nm and an emission at 530 nm. The results of intracellular ROS production were reported as a comparative percentage ratio with the control untreated group.

### 2.6. Cell Cycle Analysis

To examine the effect of metformin on cell cycle, we performed cell cycle analysis using flow cytometry as previously described [20]. In brief, SH-SY5Y cells were treated with or without 1, 5, and 10 mM metformin for 24 h in starvation culture medium. After treatment, cells were trypsinized, centrifuged at 300×g for 5 min and fixed with ice-cold 70% ethanol for 2 h. Cells were then washed with PBS and stained with Guava Cell Cycle Reagent (Millipore, Massachusetts, USA) for 30 min at room temperature in the dark. The samples were analyzed by Guava EasyCyte Plus Flow Cytometry System (Millipore, Massachusetts, USA). The results were shown as the proportion of cells in each cell cycle phase.

### 2.7. Cell Apoptosis Analysis by Flow Cytometry

To evaluate the effect of metformin on apoptotic cell death, flow cytometry was performed using Annexin V-FITC/7AAD staining (Millipore, Massachusetts, USA) to detect the percentage of cell death. SH-SY5Y cells were treated with or without 1, 5, and 10 mM metformin for 24 h. After treatment, the cells were washed with PBS, trypsinized, and stained with 100 *μ*l of Annexin V/7AAD reagent for 20 min at room temperature in the dark. The samples were analyzed by Guava EasyCyte Plus Flow Cytometry System (Millipore, Massachusetts, USA).

### 2.8. Hoechst 33342 Staining

The nuclear morphological change of apoptotic cell death was determined by Hoechst 33342 staining. SH-SY5Y cells were exposed to 1, 5, and 10 mM metformin for 24 h and then stained with Hoechst 33342 (10*μ*g/ml) in PBS at room temperature for 15 min. The apoptotic morphology of nuclei was observed and captured under inverted fluorescence microscope IX73 (model U-LH100L-3).

### 2.9. Western Blot Analysis

Whole-cell lysates were prepared from SH-SY5Y cells treated with or without 5 mM metformin (for 0, 12, and 24 h), 10 *μ*M roscovitine and 10 mM NAC in starvation culture medium using RIPA lysis buffer (ThermoFisher, Rockford, USA). After 30 min of treatment with lysis buffer on ice-cold, insoluble material was removed by centrifugation for 14,000 rpm (17,961xg) for 15 min at 4°C. The lysate protein concentration was determined using Bradford assay (Hercules, CA, USA). Equal amounts of protein were subjected to separate in SDS-PAGE and transferred to PVDF membranes (Millipore, Temecula, CA, USA). After 5% skimmed milk blocking, the immunoblots were analyzed with primary antibodies against mouse monoclonal anti-*β*-tubulin III (Santa Cruz, CA, USA), rabbit polyclonal antityrosine hydroxylase (TH) (Millipore, Massachusetts, USA), rabbit polyclonal anti-Sox6 (Santa Cruz, CA, USA), rabbit polyclonal anti-Cdk5 (Santa Cruz, CA, USA), rabbit polyclonal anti-Akt (Cell Signaling, MA, USA), rabbit polyclonal p-Akt (s473) (Cell Signaling, MA, USA), rabbit polyclonal Erk1/2 (Abcam, Cambridge, UK),and rabbit polyclonal p-Erk1/2 (pT202/pY204 for Erk1 and pT185/pY187 for Erk2) (Abcam, Cambridge, UK). Then, the membrane was incubated with horseradish peroxidase-conjugated anti-rabbit and/or anti-mouse IgG secondary antibody (Abcam, HK, USA). Immunostaining with secondary antibodies was detected using SuperSignal West Pico chemiluminescence substrate (ThermoFisher, Rockford, USA). The density of each band was quantified using Image J software and normalized with *β*-actin expression (Invitrogen, Rockford, USA). Quantitative data were reported as a comparative percentage ratio with the control untreated group.

### 2.10. Statistical Analysis

All experimental data were displayed as mean ± standard error of mean (S.E.M.). Different statistical comparisons were analyzed using pair-independent t-test compared to control group. The* p value* < 0.05 was considered as a statistically significant difference value.

## 3. Results

### 3.1. Metformin Inhibits SH-SY5Y Neuroblastoma Cell Proliferation

**Figure 1:**
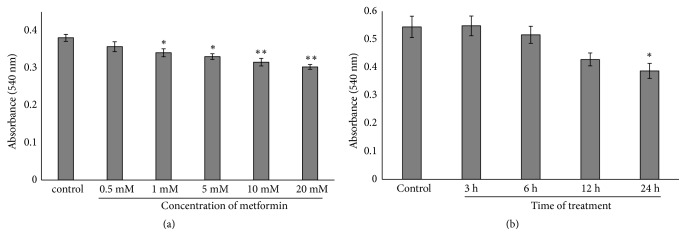
Metformin reduces cell proliferation in SH-SY5Y cells. (a) Cells were treated with various concentrations of metformin (0.5, 1, 5, 10, and 20 mM) in serum starvation culture condition for 24 h. (b) Cells were treated with 5 mM metformin in serum starvation culture condition at different times (3, 6, 12, and 24 h). Cell proliferation was determined using MTT assay. Data represented the means ± S.E.M. of three independent experiments. ^*∗*^*p*<0.05, ^*∗∗*^*p*<0.01 versus control untreated group.

To investigate the effect of metformin on SH-SY5Y cell proliferation, cells were cultured with various concentrations of metformin (0.5, 1, 5, 10, and 20 mM) for 24 h. After treatment, cell proliferation was determined using MTT assay. As shown in Figure 1(a), metformin significantly decreased cell proliferation at 1, 5, 10, and 20 mM to 89.44 ± 0.81%, 86.82 ± 0.83%, 82.86 ± 1.23%, and 79.57 ± 0.31% of the control, respectively. Next, we exposed the SH-SY5Y cells with 5 mM metformin for 3, 6, 12, and 24 h and observed that cell proliferation was significantly reduced at 24 h to 82.91 ± 2.66% of the control (Figure 1(b)).

### 3.2. Metformin Induces Cell Cycle Arrest in the G0/G1 Phase without Cell Apoptotic Induction

**Figure 2:**
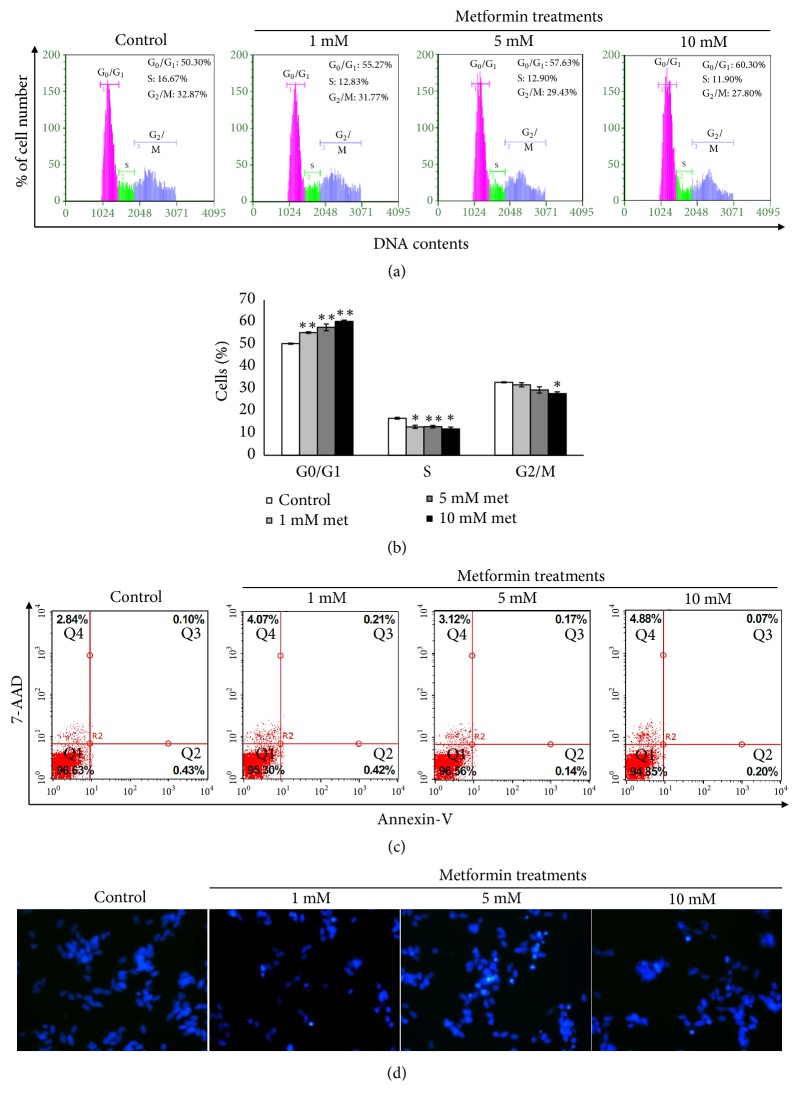
Metformin induces G0/G1 cell cycle arrest. (a) Cells were treated with or without 1, 5, and 10 mM metformin in serum starvation culture condition for 24 h. At the end of treatment, cells were fixed and stained with Guava nexin reagent for 30 min in the dark. Cell cycle was analyzed using flow cytometry. (b) The bar chart was depicted of quantitative results of cell cycle detection. Data represented the means ± S.E.M. of three independent experiments. ^*∗*^*p*<0.05, ^*∗∗*^*p*<0.01 versus control untreated group. (c) Cells were treated with or without 1, 5, and 10 mM metformin. The cells were then washed, trypsinized, and stained with Annexin V/7-AAD reagent for 20 min at room temperature. The percentage of cell apoptosis was examined using flow cytometry, (d) Apoptotic morphology of cells treated with or without 1, 5, and 10 mM metformin was stained by 10 *μ*g/ml Hoechst 33342. Met: metformin, Q1: live cells, Q2: early apoptosis, Q3: late apoptosis, and Q4: necrosis.

We further checked the cell cycle to evaluate whether metformin can inhibit cell proliferation by causing cell cycle arrest. The cell cycle phase was examined by flow cytometric analysis. The results showed that the percentage of G0/G1 phase in SH-SY5Y cells was dramatically increased following 1, 5, and 10 mM metformin treatment for 24 h to 55.27 ± 0.38%, 57.63 ± 1.51%, and 60.3 ± 0.61% of control, respectively, while S phase was significantly decreased to 12.83 ± 0.72%, 12.9 ± 0.6%, and 11.9 ± 0.87% of control, respectively (Figures 2(a) and 2(b)). To test whether cell cycle arrest was associated with apoptosis, cell apoptosis was determined using flow cytometry and Hoechst 33342 staining. The results of flow cytometric analysis indicated that treatment with 1, 5, and 10 mM metformin for 24 h did not significantly affect the level of apoptosis at* p*>0.05 (0.63 ± 0.10%, 0.31 ± 0.04% and 0.27 ± 0.07% of control, respectively) (Figure 2(c)). In addition, the morphological changes of cell apoptosis were not observed in Hoechst staining (Figure 2(d)).

### 3.3. Metformin Promotes Neuronal Differentiation and Neurite Outgrowth of SH-SY5Y Cell

**Figure 3:**
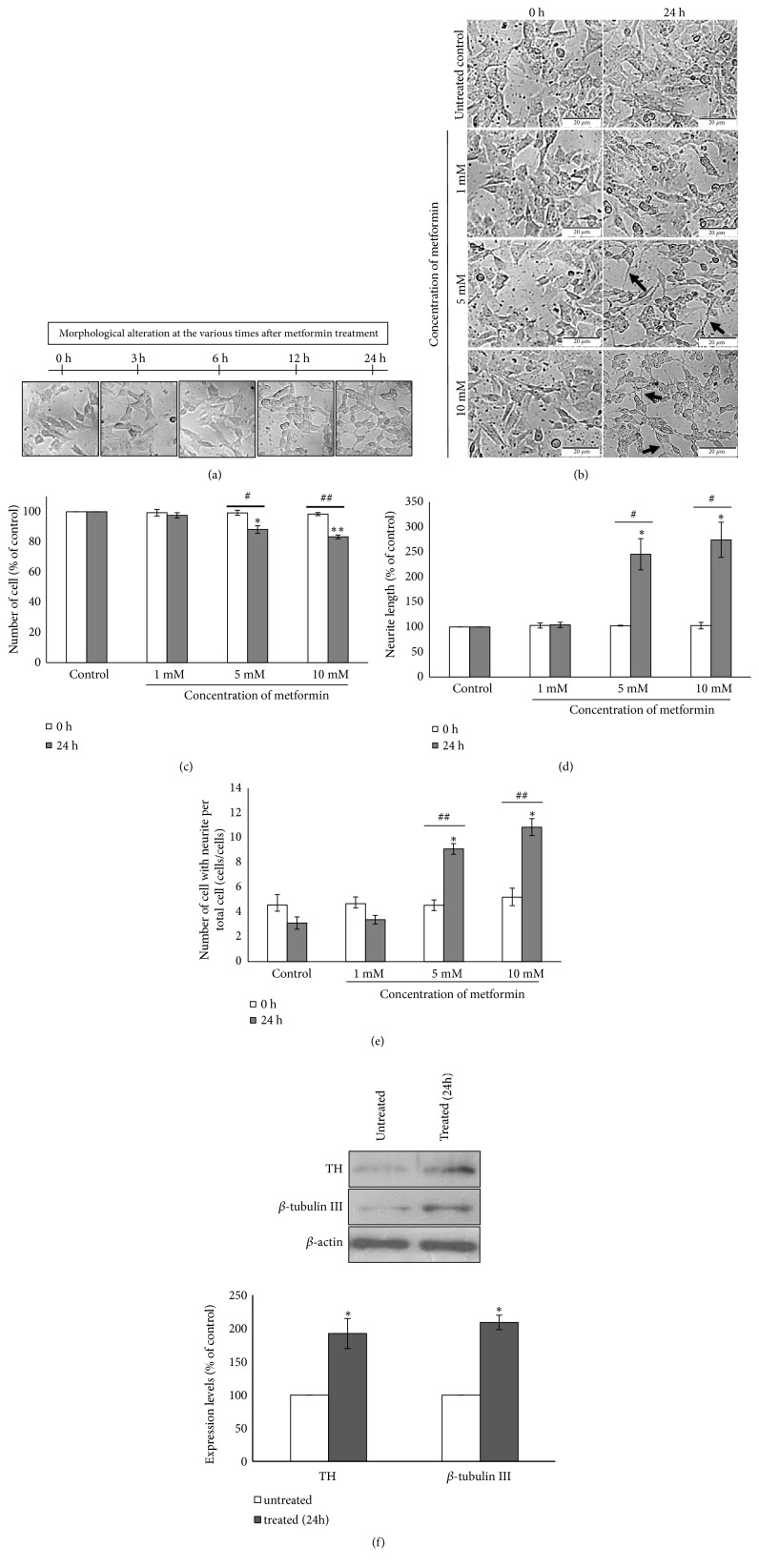
Metformin induces neuronal differentiation in SH-SY5Y cells. (a) Cell morphological alteration at various times (0, 3, 6, 12, and 24 h) after metformin exposure in serum starvation culture condition. (b) Cells morphology of SH-SY5Y cells before (0 h) and after (24 h) of 1, 5, and 10 mM metformin exposure in serum starvation culture condition. The cell morphology was observed under bright-field inverted microscope. The arrows indicated neurite outgrowth. Quantitative data of neuronal morphology was analyzed by Image J which included (c) number of cells, (d) neurite length, and (e) number of cells with neurite per total cell. (f) Cells were incubated with or without 5 mM metformin for 24 h in serum starvation culture condition. Protein expression of TH and *β*-tubulin III was determined using western blot analysis. *β*-actin was used as endogenous control. Quantitative data represented the means ± S.E.M. of three independent experiments. ^*∗*^*p*<0.05, ^*∗∗*^*p*<0.01 versus control untreated group. ^#^*p*<0.05, ^##^*p*<0.01 versus 0 h in the same treatment.

SH-SY5Y neuroblastoma cell is able to differentiate into cells with neuron-like morphology [25]. Neuronal differentiation can be facilitated by serum deprivation or serum free medium [26, 27]. In addition, it has been reported that metformin plays the potential role in inducing cell differentiation [17, 18, 28]. To examine the role of metformin in cell differentiation, SH-SY5Y cells were treated with or without 5 mM metformin in culture medium containing 1% FBS. The morphological alteration was observed and captured at the different times (0, 3, 6, 12, and 24h) after exposure to metformin. The morphological change was observed at 12 h while the neuronal morphology (neurite outgrowth) was obviously observed at 24 h (Figure 3(a)). Metformin-treated cells at the concentrations of 5 and 10 mM underwent the morphological differentiation, displaying neuron-like cells (Figure 3(b)). Furthermore, by observing the treated cells under light microscope, cell numbers were significantly decreased (Figure 3(c)) while the neurite length was dramatically increased (Figure 3(d)) and number of cells with neurite per total cell was substantively increased compared to the control (Figure 3(e)). In addition, differentiated neuronal cells were identified using the *β*-tubulin III and tyrosine hydroxylase (TH). The results revealed that metformin is significantly involved with upregulation of both neuronal markers (Figure 3(f)).

### 3.4. ROS Production Is Required for Metformin-Induced Neuronal Differentiation

**Figure 4:**
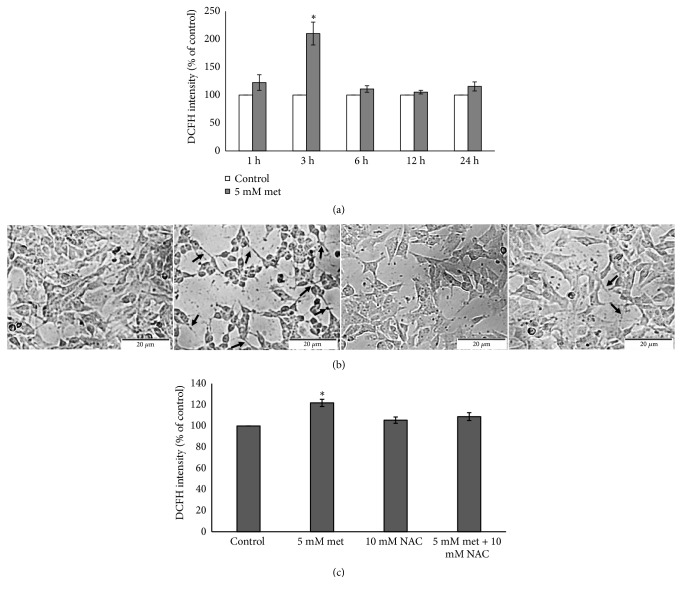
Metformin-induced neuronal differentiation is mediated by ROS production. (a) Cells were treated with or without 5 mM metformin in serum starvation culture condition for 1, 3, 6, 12, and 24 h. At the end of treatment, cells were incubated with DCFH-DA. Fluorescence intensity was detected using microplate reader. (b) Cells were treated with or without 5 mM metformin and pretreated with 10 mM NAC followed by 5 mM metformin exposure for 24 h. The cell morphology was imaged using bright-field inverted microscope. The arrow indicated neurite outgrowth. (c) Cells were pretreated with or without 10 mM NAC for 1 h followed by 5 mM metformin for 3 h. Cells were then examined for the production of ROS using DCFH-DA fluorimetric assay. Data represented the means ± S.E.M. of three independent experiments. ^*∗*^*p*<0.05, ^*∗∗*^*p*<0.01 versus control untreated group. Met: metformin, NAC: N-acetyl cysteine.

Metformin has been reported to inhibit both mitochondrial complex I and electron transfer; in that case, the generation of ROS would be expected. Therefore, we further investigated the effect of metformin on ROS production. SH-SY5Y cells were exposed to 5 mM metformin for 1, 3, 6, 12, and 24 h. At the end of incubation, intracellular ROS was measured using DCFH-DA fluorimetric assay. Fluorimetric analysis demonstrated that the intracellular ROS was significantly increased at 3 h compared with control untreated group (Figure 4(a)). ROS was reported to regulate the balances between cell growth, cell differentiation, and cell death [29]. Thus, we hypothesized that the ROS production would be required for metformin-induced neuronal differentiation. To examine this, SH-SY5Y cells were pretreated with 10 mM N-acetyl cysteine (NAC), ROS scavenger, for 1 h followed by 5 mM metformin for 24 h. The results showed that metformin remarkably induced the neurite outgrowth (arrows). However, reduction of both neurite outgrowth and neuron-like feature was observed by pretreatment with NAC (Figure 4(b)). In addition, we also examined the levels of ROS production of cells pretreated with or without 10 mM NAC for 1 h followed by 5 mM metformin for 3 h. The ROS production was significantly increased in metformin treatment while it was rescued by preincubation of NAC (Figure 4(c)). Taken together, the results suggested that ROS production would be required for metformin-induced neuronal differentiation.

### 3.5. Metformin Promotes Neuronal Differentiation through Cdk5/Sox6 Crosstalk

**Figure 5:**
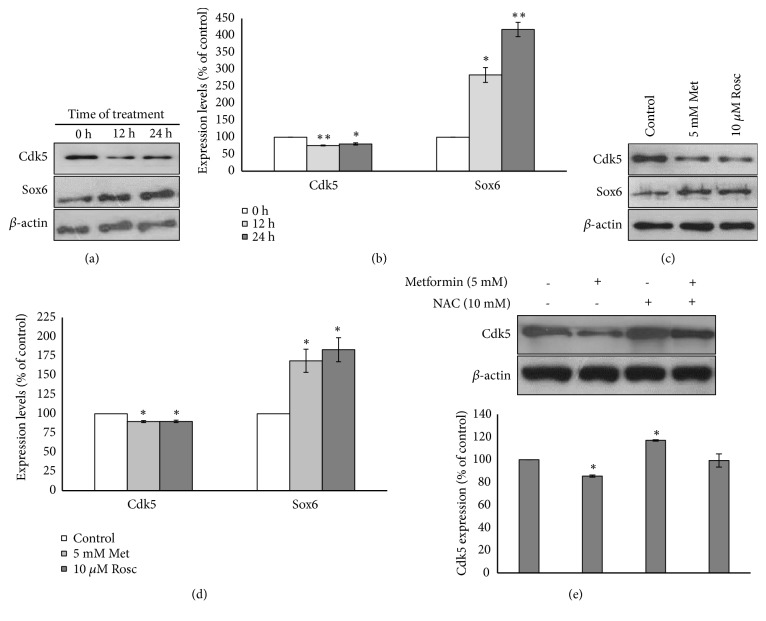
Metformin induces neuronal differentiation through cdk5/sox6 crosstalk. (a) Cells were incubated with or without 5 mM metformin for 0, 12, and 24 h in serum starvation culture condition. Protein expression of cdk5 and sox6 was determined using western blot analysis. (b) Quantitative results of Cdk5 and Sox6 expression in cells treated with or without 5 mM metformin were analyzed by Image J software. (c) Cdk5 and sox6 expression levels of cells treated with or without 5 mM metformin and 10 *μ*M roscovitine for 24 h. (d) Quantitative results of Cdk5 and Sox6 expression in cells treated with or without 5 mM metformin and 10 *μ*M roscovitine were analyzed by Image J software. (e) Cdk5 expression levels of cells treated with (+) or without (-) 5 mM metformin, in the present (+) or absent (-) of 10 mM NAC. In all western blot analysis, *β*-actin was used as endogenous control. Data represented the means ± S.E.M. of three independent experiments. ^*∗*^*p*<0.05, ^*∗∗*^*p*<0.01 versus control untreated group. Met: metformin, Rosc: roscovitine.

Cdk5 is a proline-directed serine/threonine protein kinase which is known to function in normal and pathological condition of neuronal cells [15]. Previous study reported that Cdk5 was downregulated by metformin treatment [30]. Sox6 is a transcription factor which plays an important role in neuronal development [13], including dopaminergic neuron [31]. Interestingly, Cdk5 has been documented as Sox6 suppressor involved in neuronal cell development/differentiation [14, 16]. Thus, to further investigate the mechanism underlying the role of metformin on neuronal differentiation, SH-SY5Y cells were exposed to 5 mM metformin for 12 and 24 h, prior to western blot analysis. Cdk5 protein expressions were significantly decreased while Sox6 expression levels were gradually increased compared with the control (Figures 5(a) and 5(b)). These data suggested that metformin may promote neuronal differentiation through Cdk5/Sox6 crosstalk. To strengthen this hypothesis, we treated SH-SY5Y cells with 10 *μ*M Cdk5 inhibitor roscovitine for 24 h. The results showed that expression levels of Cdk5 were significantly decreased while Sox6 expression was significantly increased compared with control untreated group (Figures 5(c) and 5(d)).

Since Cdk5 has been reported to be involved in ROS production [32], we investigated whether ROS production is associated with Cdk5 expression. Cells were pretreated with 10 mM NAC for 1 h followed by 5 mM metformin for 24 h. Cdk5 expression was determined using western blot analysis. There was no statistically significant difference between the pretreatment with NAC followed by metformin and the control untreated group (*p*>0.05). However, the expression of Cdk5 in the presence of 5 mM metformin alone was significantly downregulated (*P*<0.05). And vice versa, NAC treatment alone produced a significant upregulation of Cdk5 expression compared with control untreated group (Figure 5(e)). These data indicated that Cdk5 might be suppressed by ROS production.

### 3.6. Metformin Promotes Neuronal Differentiation via Akt and Erk1/2 Signaling Pathway

**Figure 6:**
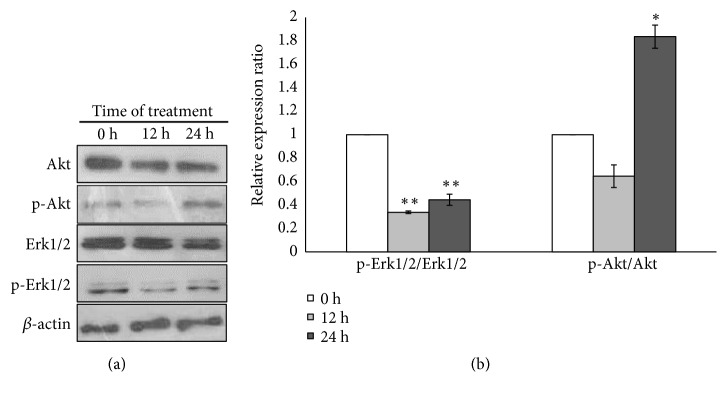
Metformin induces neuronal differentiation through Akt and Erk1/2 signaling pathway. (a) Cells were incubated with or without 5 mM metformin for 24 h in serum starvation culture condition. Protein expression of Akt, p-Akt, Erk1/2, and p-Erk1/2 was determined by western blot analysis. *β*-actin was used as endogenous control. (b) Relative expression ratios of p-Akt/Akt and p-Erk1/2/Erk1/2 were analyzed by Image J software. Data represented the means ± S.E.M. of three independent experiments. ^*∗*^*p*<0.05, ^*∗∗*^*p*<0.01 versus control untreated group.

Akt and Erk1/2 signaling cascade play an important role in the regulation of cell growth, cell survival, and cell differentiation [6, 7]. Our data revealed that metformin could promote neuronal differentiation while inhibiting cell proliferation by inducing cell cycle arrest (Figures 2–4). Further investigation whether metformin could induce neuronal differentiation via Akt and Erk1/2 signaling cascade was performed. SH-SY5Y cells were treated with or without 5 mM metformin for 24 h. Metformin decreased the phosphorylation of Erk1/2 whereas increased the phosphorylation of Akt at 24 h (Figures 6(a) and 6(b)). Therefore, the activation of Akt and inhibition of Erk1/2 may involve with the responses of SH-SY5Y cells to metformin-induced neuronal differentiation.

## 4. Discussion

According to the fact that once the primary mammalian neurons terminally differentiated into mature neuron, the cell can no longer be cultivated. To overcome this limitation, neuronal-like cell line can be a candidate for neuron-like model study. SH-SY5Y cells are, therefore, widely used as neuron-like model for studying neuronal behavior [33, 34]. There are several alternative methods to induce neuronal differentiations for the investigation of physiological functions, such as treatment with retinoic acid, brain-derived neurotrophic factor [35] and nerve growth factor [36]. Neuronal differentiations can be observed, not only from neurite growing out from each soma, but also from total length of the outgrowing neurite per soma [23]. Cell proliferation/growth arrest and differentiation exhibit a notable inverse relation [4, 24]. During cell differentiation, the proliferation usually decreased and most of cells were arrested in the G0/G1 phase [37–39]. Additionally, neuronal differentiated cells expressed the neuronal marker such as MAP2, *β*-tubulin III, NeuN, or dopaminergic neuron [2, 3, 40].

Besides being as an antidiabetic drug for treating type 2 diabetes, several reports recently showed that metformin also plays an enhancing role for cell differentiation [17, 41]. Our results showed that metformin significantly decreased cell proliferation and cell cycle was significantly higher in the G0/G1 than S and G2/M. Moreover, it also induced cell differentiation as defined by morphological alteration and the presence of *β*-tubulin III and TH, the mature neuronal markers. Cell apoptotic detection was not found in our study, although it was strongly related to cell differentiation and growth arrest/inhibition of cell proliferation [42]. However, the whole process on how metformin promotes cell differentiation still needs to be further clarified.

There is a report showed that ROS facilitates differentiation of specific cell type [9], including its determining role in cellular differentiation [43]. The role of ROS in the regulation of neuronal differentiation also highlighted* in vitro* approaches using cells derived from neuroblastoma cell line [44]. In neuron, the ROS scavengers suppressed neurosphere formation [45]. Increase of neuronal differentiation was related to the metabolic pathway and ROS production [10]. When cells were exposed to metformin, our result revealed the enhancement of ROS production at 3 h, together with the changes of cell morphology into a differentiated form. On the other hand, the neurite outgrowth was decreased in the present of pretreatment of NAC. Thus, our present study indicated that ROS should involve in metformin-induced SH-SY5Y differentiation.

Interestingly, our result noted that metformin downregulated Cdk5 while preincubation with NAC increased Cdk5 expression level. Cdk5 was related to both normal neuronal development and neurodegeneration [46]. Cdk5 is activated by its specific activators, p35 or p25. Cdk5 controls the final proliferation/differentiation switch during the neuronal development. Additionally, several evidences suggested that Cdk5 appeared favourable in maintaining a quiescent state of neurons during its development [47, 48]. Although Cdk5 is highly activated in cancer, its function is still elusive. Previous study reported that Cdk5 contributes to cancer proliferation, migration, and chemotherapy resistance [49]. It has been reported that Cdk5 modulated retinoblastoma (Rb)/E2F pathway, resulting in promotion of G0/G1 to S phase transition and initiation of cell cycle [48]. Our results corresponded to the previous study that metformin may inhibit cell cycle in G0/G1 phase via downregulation of Cdk5 in neuroblastoma. By the way, ROS not only participate in the chemical damage of cell components but also are involved in maintaining of cell redox homeostasis and signaling pathway. ROS can promote either survival or apoptosis depending on their concentration and type of cancer cell [50]. Metformin increased ROS levels in HCT116 and HCT116 p53^−/−^ cells, but not in HT29 cells, leading to inducing cell cycle arrest [51]. However, the link between ROS production and Cdk5 level has not yet been fully elucidated. Cdk5 was previously reported to localize in the inner membrane of mitochondria which regulated mitochondrial depolarization and level of ROS. In fact, in neurons, ROS is strongly related to Cdk5 by which induction of Cdk5 happens through p25 activator in response to activating apoptotic cell death, especially in neurodegenerative disorder [52]. Our model used SH-SY5Y cells which are cancer/tumor cells; however, the complicated roles of Cdk5 in cancer, its upregulated expression, including the function of p25 activator, have not yet well understood. Recently, it has been revealed that loss of Cdk5 in cancer increased the production of mitochondrial ROS [32]. Thus, one possibility is that metformin mediated ROS may have a retrograde effect to suppress Cdk5 expression.

Previous study suggested that Sox 6 was evaluated as a direct nuclear target of Cdk5 which was phosphorylated by Cdk5/p35 [16]. Sox6 is a transcription factor which plays a pivotal role in development and involves in multiple physiological processes [13]. Sox6 is expressed in differentiating neuron, observed from expression in Tuj positive neuron. To answer the question whether Sox6 is related to metformin-induced neuronal differentiation via Cdk5 or not, the experiment with Cdk5 inhibitor, roscovitine, was performed. Our data showed that in the presence of roscovitine, Sox 6 protein expression level was significantly increased, in contrast with the expression of Cdk5. These demonstrated that Cdk5 has a relation, crosstalk, with Sox6 in metformin promoted cell differentiation.

The Erk1/2 and Akt signaling pathways play an important role in cell proliferation, growth, and differentiation [6, 7]. Our results indicated that metformin dramatically reduced Erk1/2 phosphorylation while activating Akt phosphorylation during neuronal differentiation. It might be possible that metformin may have an allosteric mTOR inhibition in response to indirect activation of AMPK signaling. These effects increase Akt phosphorylation whereas decrease Erk1/2 phosphorylation which agree with a recent study, using rapamycin as allosteric inhibition of mTOR [22]. Inhibition of Erk1/2 phosphorylation is involved in growth arrest which is consistent with the results of cell cycle, cell proliferation and reduction of Erk1/2 phosphorylation during metformin induction of neuronal differentiation. It has been reported that Erk1/2 has two effects which are kinase-dependent and -independent effect. Regulation and function of both effects of Erk1/2 are obviously different [53]. Kinase-dependent effect of Erk1/2 controls cell proliferation by phosphorylation of p21^cip1^, a Cdks inhibitor, resulting in induction of degradation of p21^cip1^ via proteasome and ubiquitination. In contrast, kinase-independent effect of Erk1/2 regulates growth arrest through suppression of p21^cip1^ phosphorylation in both normal cells and cancer cells [54]. On the other hand, increasing of Akt phosphorylation is related to neuronal differentiation. Induction of neuronal differentiation requires Akt activation for upregulating neuron-specific gene neurogenin 1 [55]. Thus, Erk1/2 inhibition and Akt activation by metformin treatment might mediate neuronal differentiation and growth arrest in neuroblastoma.

Of note, our results showed that metformin exposure did not only change phosphorylated expression levels of Akt and Erk1/2 but also alter their total expression levels. This may be because of phosphorylation sites of Akt at* Ser473*, which cause a decrease of Akt to prevent the hyperphosphorylation of Akt. Then, degradation of Akt was activated by proteasome and ubiquitin [56]. On the other hand, Erk1/2 was phosphorylated within the activation loop on both residues* Thr202* and* Tyr204* [57]. Despite Erk1/2 which was required for controlling cell differentiation and survival, the underlying mechanism regulating the activation of Erk1/2 is still elusive. Our study showed that the levels of total Erk1/2 were decreased. A reduction of Erk1/2 phosphorylation of both residues might lead to a decrease of Erk1/2.

The present study demonstrated that metformin promotes neuronal differentiation by the reduction of cellular proliferation and induction of ROS. Analysis of the mechanism underlying these changes indicated that metformin produces neuronal differentiation via the crosstalk between Cdk5/Sox6 and may relate to Erk1/2 and Akt signaling. Taken together, this investigation could provide a basis strategy for the development of neurodegenerative disease treatment.

## 5. Conclusions

**Figure 7:**
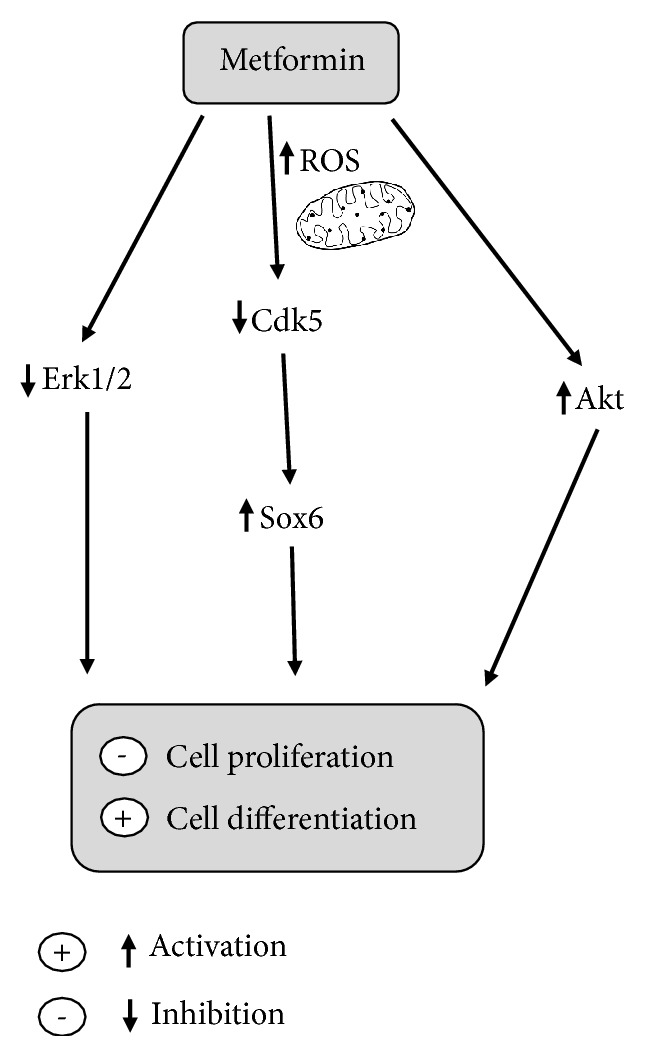
Proposed mechanisms by which metformin induces neuronal differentiation and inhibits cell proliferation.

The present study demonstrated that metformin promotes neuronal differentiation by the reduction of cellular proliferation and induction of ROS. In addition, Cdk5 may be influenced by ROS because the ROS was produced earlier at 3 h after treatment and decline after all, while Cdk5 was significantly decreased at 12 h. Further examination revealed that metformin significantly reduced Cdk5 expression while increased Sox6 expression levels. As Sox6 is a target of Cdk5, this result may indicate a role in crosstalk of Cdk5 and Sox6 during neuronal differentiation. Last but not least, the results noted that metformin induces phosphorylated Akt but decreases phosphorylated Erk1/2.Taken together, metformin induces neuronal differentiation through Cdk5/Sox6, including Erk1/2 and Akt signaling pathway (Figure 7). These informative data could apply for the idea of using metformin as a treatment strategy for neurodegenerative disease.

## Data Availability

The data used to support the findings of this study are included within the article.

## References

[1] Sell S. (2006). Cancer stem cells and differentiation therapy. *Tumor Biology*.

[2] Lopes F. M., Schröder R., Júnior M. L. C. D. F. (2010). Comparison between proliferative and neuron-like SH-SY5Y cells as an in vitro model for Parkinson disease studies. *Brain Research*.

[3] Cheung Y.-T., Lau W. K.-W., Yu M.-S. (2009). Effects of all-*trans*-retinoic acid on human SH-SY5Y neuroblastoma as *in vitro* model in neurotoxicity research. *NeuroToxicology*.

[4] Ruijtenberg S., van den Heuvel S. (2016). Coordinating cell proliferation and differentiation: antagonism between cell cycle regulators and cell type-specific gene expression. *Cell Cycle*.

[5] Lange C., Calegari F. (2010). Cdks and cyclins link G1 length and differentiation of embryonic, neural and hematopoietic stem cells. *Cell Cycle*.

[6] Gharibi B., Ghuman M. S., Hughes F. J. (2012). Akt- and Erk-mediated regulation of proliferation and differentiation during PDGFR *β*-induced MSC self-renewal. *Journal of Cellular and Molecular Medicine*.

[7] Asati V., Mahapatra D. K., Bharti S. K. (2016). PI3K/Akt/mTOR and Ras/Raf/MEK/ERK signaling pathways inhibitors as anticancer agents: Structural and pharmacological perspectives. *European Journal of Medicinal Chemistry*.

[8] Nugud A., Sandeep D., El-Serafi A. T. (2018). Two faces of the coin: minireview for dissecting the role of reactive oxygen species in stem cell potency and lineage commitment. *Journal of Advanced Research*.

[9] Ji A.-R., Ku S.-Y., Cho M. S. (2010). Reactive oxygen species enhance differentiation of human embryonic stem cells into mesendodermal lineage. *Experimental & Molecular Medicine*.

[10] Moliner A., Enfors P., Ibáñez C. F., Andäng M. (2008). Mouse embryonic stem cell-derived spheres with distinct neurogenic potentials. *Stem Cells and Development*.

[11] Sart S., Song L., Li Y. (2015). Controlling redox status for stem cell survival, expansion, and differentiation. *Oxidative Medicine and Cellular Longevity*.

[12] Visweswaran M., Pohl S., Arfuso F. (2015). Multi-lineage differentiation of mesenchymal stem cells–To Wnt, or not Wnt. *International Journal of Biochemistry & Cell Biology*.

[13] Connor F., Wright E., Denny P., Koopman P., Ashworth A. (1995). The Sry-related HMG box-containing gene Sox6 is expressed in the adult testis and developing nervous system of the mouse. *Nucleic Acids Research*.

[14] Batista-Brito R., Rossignol E., Hjerling-Leffler J. (2009). The cell-intrinsic requirement of Sox6 for cortical interneuron development. *Neuron*.

[15] Tsai L.-H., Takahashi T., Caviness V. S., Harlow E. (1993). Activity and expression pattern of cyclin-dependent kinase 5 in the embryonic mouse nervous system. *Development*.

[16] Rudrabhatla P., Utreras E., Jaffe H., Kulkarni A. B. (2014). Regulation of Sox6 by cyclin dependent kinase 5 in brain. *PLoS ONE*.

[17] Qin W., Gao X., Ma T. (2018). Metformin enhances the differentiation of dental pulp cells into odontoblasts by activating AMPK signalling. *Journal of Endodontics*.

[18] Ahn M.-J., Cho G.-W. (2017). Metformin promotes neuronal differentiation and neurite outgrowth through AMPK activation in human bone marrow–mesenchymal stem cells. *Biotechnology and Applied Biochemistry*.

[19] Dadwal P., Mahmud N., Sinai L. (2015). Activating endogenous neural precursor cells using metformin leads to neural repair and functional recovery in a model of childhood brain injury. *Stem Cell Reports*.

[20] Wang Y., Xu W., Yan Z. (2018). Metformin induces autophagy and G0/G1 phase cell cycle arrest in myeloma by targeting the AMPK/mTORC1 and mTORC2 pathways. *Journal of Experimental & Clinical Cancer Research*.

[21] Rodríguez-Lirio A., Pérez-Yarza G., Fernández-Suárez M. R., Alonso-Tejerina E., Boyano M. D., Asumendi A. (2015). Metformin induces cell cycle arrest and apoptosis in drug-resistant leukemia cells. *Leukemia Research and Treatment*.

[22] Soares H. P., Ni Y., Kisfalvi K., Sinnett-Smith J., Rozengurt E. (2013). Different patterns of Akt and ERK feedback activation in response to rapamycin, active-site mTOR inhibitors and metformin in pancreatic cancer cells. *PLoS ONE*.

[23] Fadó R., Moubarak R. S., Miñano-Molina A. J. (2013). X-linked inhibitor of apoptosis protein negatively regulates neuronal differentiation through interaction with cRAF and Trk. *Scientific Reports*.

[24] Chen M.-C., Lin H., Hsu F.-N., Huang P.-H., Lee G.-S., Wang P. S. (2010). Involvement of cAMP in nerve growth factor-triggered p35/Cdk5 activation and differentiation in PC12 cells. *American Journal of Physiology-Cell Physiology*.

[25] Gao X., Joselin A. P., Wang L. (2010). Progranulin promotes neurite outgrowth and neuronal differentiation by regulating GSK-3*β*. *Protein & Cell*.

[26] Evangelopoulos M. E., Weis J., Krüttgen A. (2005). Signalling pathways leading to neuroblastoma differentiation after serum withdrawal: HDL blocks neuroblastoma differentiation by inhibition of EGFR. *Oncogene*.

[27] Tremblay R. G., Sikorska M., Sandhu J. K., Lanthier P., Ribecco-Lutkiewicz M., Bani-Yaghoub M. (2010). Differentiation of mouse Neuro 2A cells into dopamine neurons. *Journal of Neuroscience Methods*.

[28] Mu W., Wang Z., Ma C. (2018). Metformin promotes the proliferation and differentiation of murine preosteoblast by regulating the expression of sirt6 and oct4. *Pharmacological Research*.

[29] Bigarella C. L., Liang R., Ghaffari S. (2014). Stem cells and the impact of ROS signalling. *Development*.

[30] Wallbillich J. J., Josyula S., Saini U. (2017). High glucose-mediated STAT3 activation in endometrial cancer is inhibited by metformin: therapeutic implications for endometrial cancer. *PLoS ONE*.

[31] Panman L., Papathanou M., Laguna A. (2014). Sox6 and Otx2 control the specification of substantia nigra and ventral tegmental area dopamine neurons. *Cell Reports*.

[32] Navaneethakrishnan S., Rosales J. L., Lee K.-Y. (2018). Loss of Cdk5 in breast cancer cells promotes ROS-mediated cell death through dysregulation of the mitochondrial permeability transition pore. *Oncogene*.

[33] Costa D., Gigoni A., Würth R., Cancedda R., Florio T., Pagano A. (2014). Metformin inhibition of neuroblastoma cell proliferation is differently modulated by cell differentiation induced by retinoic acid or overexpression of NDM29 non-coding RNA. *Cancer Cell International*.

[34] Le M. T. N., Xie H., Zhou B. (2009). MicroRNA-125b promotes neuronal differentiation in human cells by repressing multiple targets. *Molecular and Cellular Biology*.

[35] Kaplan D. R., Matsumoto K., Lucarelli E., Thielet C. J. (1993). Induction of TrkB by retinoic acid mediates biologic responsiveness to BDNF and differentiation of human neuroblastoma cells. *Neuron*.

[36] Chen J., Chattopadhyay B., Venkatakrishnan G., Ross A. H. (1990). Nerve growth factor-induced differentiation of human neuroblastoma and neuroepithelioma cell lines.. *Cell growth & differentiation : the molecular biology journal of the American Association for Cancer Research*.

[37] Goda H., Sakai T., Kurosumi M., Inoue K. (1998). Prolactin-producing cells differentiate from G0/G1-arrested somatotrophs in vitro: an analysis of cell cycle phases and mammotroph differentiation. *Endocrine Journal*.

[38] Akiba J., Murakami Y., Noda M. (2011). N-myc downstream regulated gene1/Cap43 overexpression suppresses tumor growth by hepatic cancer cells through cell cycle arrest at the G0/G1 phase. *Cancer Letters*.

[39] Fragale A., Tartaglia M., Bernardini S. (1999). Decreased proliferation and altered differentiation in osteoblasts from genetically and clinically distinct craniosynostotic disorders. *The American Journal of Pathology*.

[40] Encinas M., Iglesias M., Liu Y. (2000). Sequential treatment of SH‐SY5Y cells with retinoic acid and brain‐derived neurotrophic factor gives rise to fully differentiated, neurotrophic factor‐dependent, human neuron‐like cells. *Journal of Neurochemistry*.

[41] Wang P., Ma T., Guo D. (2018). Metformin induces osteoblastic differentiation of human induced pluripotent stem cell‐derived mesenchymal stem cells. *Journal of Tissue Engineering and Regenerative Medicine*.

[42] Vieira Torquato H. F., Ribeiro-Filho A. C., Buri M. V. (2017). Canthin-6-one induces cell death, cell cycle arrest and differentiation in human myeloid leukemia cells. *Biochimica et Biophysica Acta (BBA) - General Subjects*.

[43] Sohal R. S., Allen R. G., Nations C. (1986). Oxygen free radicals play a role in cellular differentiation: an hypothesis. *Journal of Free Radicals in Biology & Medicine*.

[44] Vieira H. L. A., Alves P. M., Vercelli A. (2011). Modulation of neuronal stem cell differentiation by hypoxia and reactive oxygen species. *Progress in Neurobiology*.

[45] Yoneyama M., Kawada K., Gotoh Y., Shiba T., Ogita K. (2010). Endogenous reactive oxygen species are essential for proliferation of neural stem/progenitor cells. *Neurochemistry International*.

[46] Dhavan R., Tsai L.-H. (2001). A decade of CDK5. *Nature Reviews Molecular Cell Biology*.

[47] Kawauchi T. (2014). Cdk5 regulates multiple cellular events in neural development, function and disease. *Development, Growth & Differentiation*.

[48] Futatsugi A., Utreras E., Rudrabhatla P., Jaffe H., Pant H. C., Kulkarni A. B. (2012). Cyclin-dependent kinase 5 regulates E2F transcription factor through phosphorylation of Rb protein in neurons. *Cell Cycle*.

[49] Pozo K., Bibb J. A. (2016). The emerging role of Cdk5 in cancer. *Trends in Cancer*.

[50] Biswas S., Chida A. S., Rahman I. (2006). Redox modifications of protein–thiols: emerging roles in cell signaling. *Biochemical Pharmacology*.

[51] Mogavero A., Maiorana M. V., Zanutto S. (2017). Metformin transiently inhibits colorectal cancer cell proliferation as a result of either AMPK activation or increased ROS production. *Scientific Reports*.

[52] Fitzgerald J. C., Camprubi M. D., Dunn L. (2012). Phosphorylation of HtrA2 by cyclin-dependent kinase-5 is important for mitochondrial function. *Cell Death & Differentiation*.

[53] Park J.-I. (2014). Growth arrest signaling of the Raf/MEK/ERK pathway in cancer. *Frontiers in Biology*.

[54] Guégan J.-P., Ezan F., Gailhouste L., Langouët S., Baffet G. (2014). MEK1/2 overactivation can promote growth arrest by mediating ERK1/2‐dependent phosphorylation of p70S6K. *Journal of Cellular Physiology*.

[55] Zhang X., He X., Li Q. (2017). PI3K/AKT/mTOR signaling mediates valproic acid-induced neuronal differentiation of neural stem cells through epigenetic modifications. *Stem Cell Reports*.

[56] Wu Y.-T., Ouyang W., Lazorchak A. S., Liu D., Shen H.-M., Su B. (2011). mTOR complex 2 targets Akt for proteasomal degradation via phosphorylation at the hydrophobic motif. *The Journal of Biological Chemistry*.

[57] Lai S., Pelech S. (2016). Regulatory roles of conserved phosphorylation sites in the activation T-loop of the MAP kinase ERK1. *Molecular Biology of the Cell (MBoC)*.

